# Management of Anterior Abdominal Wall Defect Using a Pedicled Tensor Fascia Lata Flap: A Case Report

**DOI:** 10.1155/2012/487126

**Published:** 2012-11-25

**Authors:** K. D. Ojuka, F. Nangole, M. Ngugi

**Affiliations:** Department of Surgery, College of Health Sciences, University of Nairobi, P.O. Box 19676, Nairobi 00202, Kenya

## Abstract

Degloving injuries to anterior abdominal wall are rare due to the mechanism of injury. Pedicled tensor fascia lata is known to be a versatile flap with ability to reach the lower anterior abdomen. A 34-year-old man who was involved in a road traffic accident presented with degloving injury and defect at the left inguinal region, sigmoid colon injury, and scrotal bruises. At investigation, he was found to have pelvic fracture. The management consisted of colostomy and tensor fascia lata to cover the defect at reversal. Though he developed burst abdomen on fifth postoperative day, the flap healed with no complications.

## 1. Background

Degloving injuries of the anterior abdominal wall are rare. This is due to the fact that pathophysiological basis for degloving injury is shear forces that avulses the skin and subcutaneous tissue from the underlying tissues. These forces result from parallel and counteractive forces generated against external force, when counteractive forces are produced by the underlying solid framework. The perforating vessels underneath are disrupted; as a consequence, vascularity of the skin is compromised [1–5]. As a result, most degloving injuries are seen at areas supported by the underlying skeletal framework, mostly the extremities. 

As a pedicled flap, tensor fascia lata is versatile; its reach to the lower abdomen and groin has made it an attractive option for reconstructing soft tissue defects. However, debate exists on the safe dimension of the flap, as distal tip necrosis is common. Also, the adequacy of the fascia lata as a sole substitute for abdominal wall muscles has its controversies [6]. 

## 2. Objective

We report a case of degloving injury of the anterior abdominal wall following an accident, managed by pedicled tensor fascia lata flap.

### 2.1. Case

**Figure 1:**
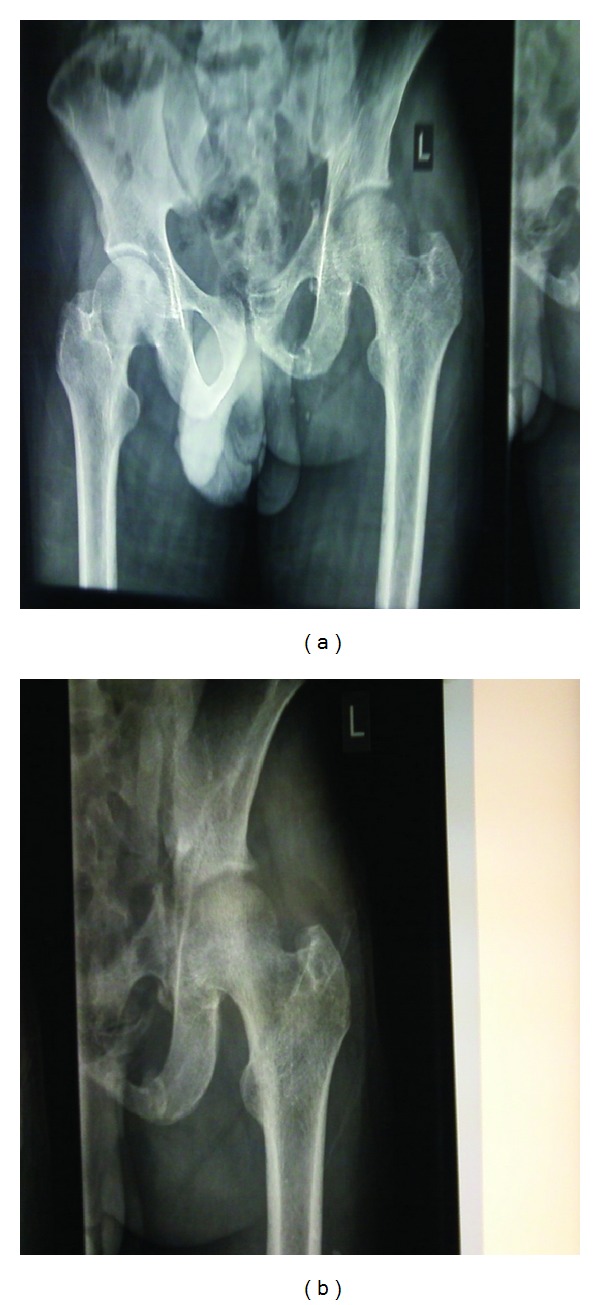
Pelvic injury.

**Figure 2:**
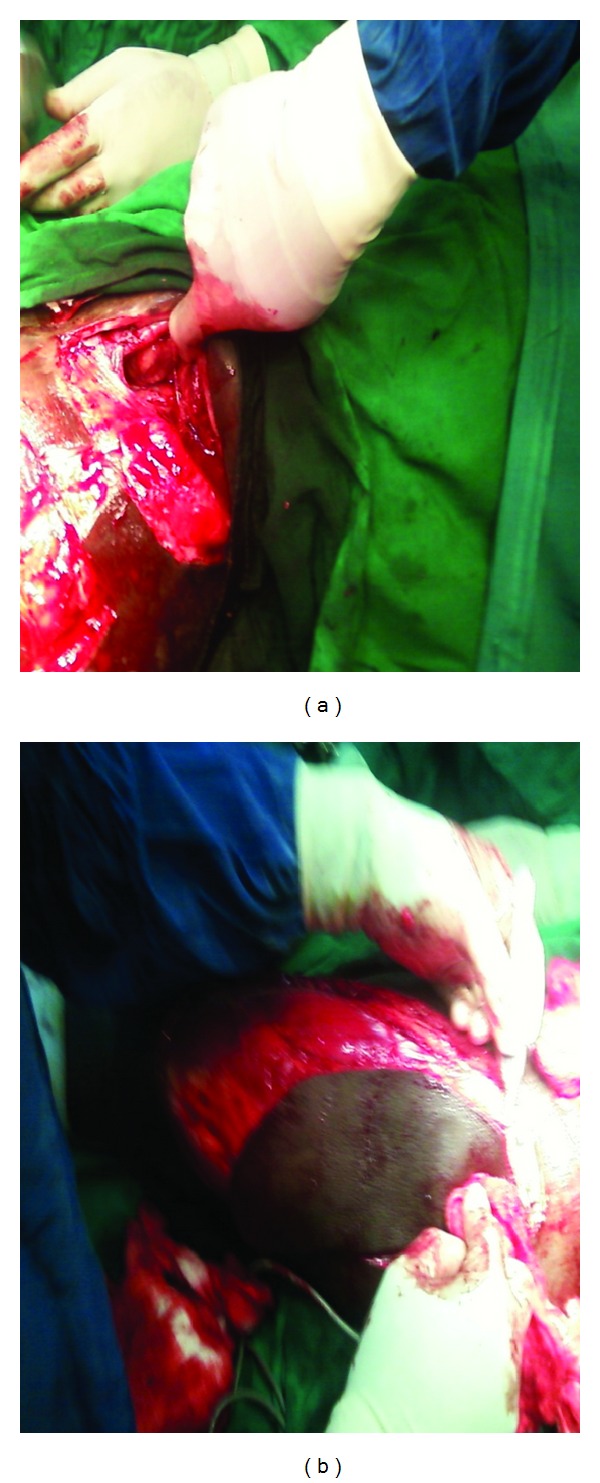
Defect and flap.

**Figure 3:**
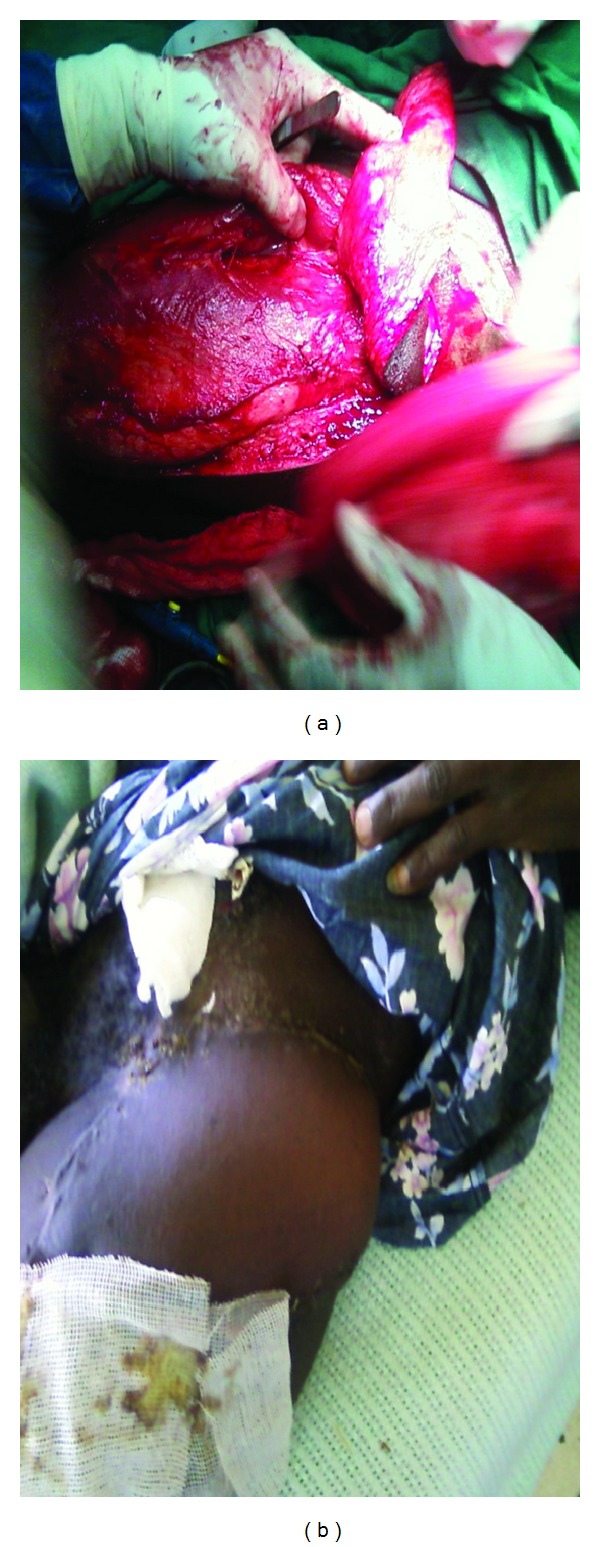
The pedicle and healing flap and graft.

A 34-year-old man was referred to our surgical unit from a cottage hospital on the day he was involved in a road traffic accident as a pedestrian. He was going to work when he was hit by an overlapping van along the road. He fell down and the van ran over his left thigh and left inguinal area. He lost consciousness and found himself at a private hospital where initial resuscitation was performed and he was transferred to Kenyatta National Hospital. On examination he was found to be dehydrated, had peritonitis, degloving injury over the left inguinal area, and bruised perineum. Radiological investigations revealed pelvic fracture (Figure 1). He was optimized and laparotomy was performed a day after admission. Intraoperatively, the sigmoid colon was found to be bruised and perforated, 10 cm of which were resected and Hartman's colostomy performed. Debridement of the injured left inguinal abdominal wall left a 5 cm by 10 cm defect (Figure 2(a)). An attempt was made to stitch it to the skin over the thigh. On the third postoperative day the attempted stitching gave way due to infection. The patient also started to bleed on an off from the pelvic fracture through the defect. The bleeding was managed by tranexamic and packing and later stopped by the tenth postoperative day. The wound was managed by daily dressing until it was well granulated by the fifth month after the injury. Decision was made to reverse the colostomy and close the abdominal wall defect at the same time to save time and money. Colostomy reversal and pedicled tensor fascia lata flap were used to close the defect in the anterior abdominal wall defect. Split thickness skin graft was used from the same thigh to close the other defect. On the fifth postoperative day he developed burst abdomen. This was repaired a day later after optimization and healed well. The flap and the graft took well (Figure 3(b)). He has been discharged and is on follow up in the surgical outpatient clinic with no complications.

## 3. Discussion 

Traumatic loss of the abdominal wall presents a difficult reconstructive challenge. The reconstruction of anterior abdominal wall defect requires an understanding of the anatomy of both the anterior abdominal wall and lower extremity [6]. The defect ranges from partial-thickness defect to full-thickness wound. A full thickness defect includes loss of both superficial soft tissue and the deeper musculofascial layers [7].

The reconstruction of the defect will then range from graft, tissue expanders, primary closure, and negative pressure closures to flaps depending on the degree of defect. Even among the flaps, the use depends on the location and size of the defect though the most used are pedicled [7–9].

The commonly used loco regional flaps include but are not limited to external oblique muscle, tensor fascia lata, rectus abdominis, rectus femoris, anterolateral thigh, latissimus dorsi, and omental flaps. Most of them will require the combination with bioprosthetic or synthetic mesh that allows for the recapitulation of abdominal wall form and function by the surgeon [6].

Tensor fascia lata flap was first described by Wangensteen in 1934 for abdominal wall reconstruction [6]. This flap started to gain popularity after further description by Nahai et al., 1978 and 1979 [10, 11]. It is a versatile flap that is regarded as more useful in anterior abdominal wall defects that are in the lower third. This is because the thickness of the fascia provides the strength required in reconstructing the area and therefore excludes the need for underlying mesh as required in the other flaps. Caution is however required for the unreliability of the distal one third of the skin paddle [6]. This has been demonstrated by this case where the healing has occurred well and no problem has been noticed with regard to strength and form of the anterior abdominal wall.
